# Excellent clinical outcomes of renal transplant from pediatric deceased donors with acute kidney injury

**DOI:** 10.1186/s40001-023-01111-9

**Published:** 2023-05-04

**Authors:** Qiuhao Liu, Hedong Zhang, Mingda Zhong, Liang Tan, Shanbiao Hu, Longkai Peng, Xubiao Xie, Gongbin Lan

**Affiliations:** 1 Department of Kidney Transplantation, The Second Xiangya Hospital of Central South University, No. 139 Renmin Road, Changsha, 410011 Hunan Province China; 2 Clinical Research Center for Organ Transplantation in Hunan Province, Changsha, China; 3 Clinical Immunology Center, Central South University, Changsha, China

**Keywords:** Kidney transplantation, Acute kidney injury, Pediatric, Clinical outcome, Delayed graft function

## Abstract

**Background:**

The use of kidneys from deceased donors with acute kidney injury (AKI) to expand the donor pool is an ongoing trend. Prior research on the utilization of AKI donor kidneys, especially from pediatric AKI donors, was limited and has been subject to small sample sizes. In this study, we aimed to evaluate the safety and effectiveness of early post-transplantation outcomes in pediatric deceased donors with AKI.

**Methods:**

This retrospective study compared the clinical results (including delayed graft function [DGF], acute rejection, patient and death-censored graft survival rates and renal function post-transplant) of kidney transplantation from deceased donors who were categorized as pediatric donors and adult donors with or without AKI, as defined by the Kidney Disease: Improving Global Outcomes (KIDGO) criteria, at our center between January 2018 and December 2020.

**Results:**

Of the 740 patients, 154 received kidneys from pediatric donors (with AKI group [n = 41]; without AKI group [n = 113]), and 586 received kidneys from adult donors (with AKI group [n = 218]; without AKI group [n = 368]). The baseline characteristics were similar in both cohorts. No significant difference was observed in 1-year patient survival, death-censored graft survival, or acute rejection between the AKI and non-AKI groups in both the pediatric and adult cohorts. However, compared with those transplanted with adult AKI kidneys, those transplanted with pediatric AKI kidneys showed a superior recovery of allograft function. In pediatric cohorts, no significant difference was found in serum creatinine/estimated glomerular filtration rate (SCr/eGFR) between the AKI and non-AKI groups, even in the first week post-transplant. In contrast, the post-transplant SCr/eGFR level of the AKI group recipients in adult cohorts did not recover to a level statistically similar to that of non-AKI recipients, even at 6-months post-transplant. Nonetheless, AKI kidney recipients were at an increased risk of DGF in both pediatric (34.1% vs. 16.8%) and adult (38.5% vs. 17.4%) cohorts.

**Conclusions:**

Kidney transplantation from deceased donors with AKI has short-term clinical outcomes comparable to those of non-AKI kidney transplantation. Pediatric AKI kidneys have a superior recovery of allograft function. The transplant community should utilize this donor pool to minimize waiting-list-related mortalities.

## Introduction

Kidney transplantation is the preferred choice for patients with chronic kidney disease (CKD) and end-stage renal disease (ESRD), owing to improvements in long-term survival and quality of life when compared with maintenance dialysis [1, 2]. However, the limited supply of high-quality deceased donor kidneys has long been unable to meet the rising demand for kidney transplantations [3, 4]. As a result, it has led to increased use of marginal kidneys, including kidneys with acute kidney injury (AKI) to expand the donor pool [5, 6].

AKI is a syndrome characterized by the rapid loss of renal excretory function and can be diagnosed by decreased urine output and/or the accumulation of end products of nitrogen metabolism [7, 8]. AKI is thought to be associated with CKD [9, 10] and is often considered a reversible functional renal impairment, which is mainly characterized by acute tubular necrosis (ATN) [8, 11]. Previous multicenter reviews based on deceased donor kidney transplant cohort studies have found that donors with AKI and other known risk factors are not associated with long-term all-cause graft failure [12, 13]. Nonetheless, debate has continued regarding the use of deceased donors with AKI. Several studies have reported worse clinical outcomes when deceased donor kidneys with AKI were used [14, 15]. When considering donors, AKI can activate body repair processes and initiate ischemic preconditioning, which can be beneficial for graft function repair in the recipient [4, 16], and the kidneys procured from pediatric deceased donors may have greater repair potential because the donors are younger, with fewer underlying disease or comorbidities [17, 18]. However, there are scant data and allocation practices on the results of pediatric AKI deceased donor kidney transplantation, and the reliability of pediatric donors with AKI remains controversial [17, 18]. Therefore, in this study, we aimed to evaluate the safety and effectiveness of early post-transplantation outcomes in pediatric deceased donors with AKI under various circumstances.

## Materials and methods

### Patient cohort and clinical data

This was a retrospective, single-center cohort study of patients who received deceased donor kidney transplants between January 2018 and December 2020. All study data were obtained from the China Scientific Registry of Kidney Transplantation (CSRKT) and the China Organ Transplant Response System (COTRS). The study was performed in accordance with the Declaration of Helsinki, Istanbul declaration standards, and the principles of Good Clinical Practice. All cases of organ donation and transplantation met the Chinese standards for human organ donation [19]. The study procedures were reviewed and approved by the Ethics Committee of the Second Xiangya Hospital of the Central South University.

All recipients were divided into four groups according to donor age (pediatric, < 18 years; adult, ≥ 18 years) and donor AKI status (with or without AKI). AKI was defined using the Kidney Disease: Improving Global Outcomes (KIDGO) criteria [20, 21]: an increase in SCr of 0.3 mg/dL (divided by 88.4 µmol/L to convert SCr level to micromoles per liter) within 48 h or an increase in SCr level to 1.5 times the baseline within the prior 7 days, irrespective of the urine output, as it was not available in the data set. AKI stage was defined using the KDIGO SCr level criteria as follows [20]: stage 1 (0.3 mg/dL or 50% increase from admission to the terminal SCr level), stage 2 (100% increase from admission to the terminal SCr level), and stage 3 (> 4.0 mg/dL or 200% increase from admission to the terminal SCr level), irrespective of urine output or dialysis initiation, as these data were not available. The exclusion criteria were ABO incompatibility, re-transplant recipients, or patients who underwent double kidney transplantation (including en-bloc or separate double kidney transplants).

All recipients received mycophenolate mofetil (MMF; 1 g) and intravenous methylprednisolone (500 mg) before transplantation. Basiliximab (more in non-AKI group) or antithymocyte globulin was used as induction therapy, and tacrolimus, MMF, and methylprednisolone were administered after kidney transplantation. The minimum concentration of tacrolimus was maintained at 7–10 ng/mL during the first 3 months and at 6–8 ng/mL during the first year post-transplantation. MMF was administered at an oral dose of 0.75 g twice daily, and the MMF area under the curve was maintained at 30–60 mg·h/L. Following intravenous methylprednisolone (1.5 g), oral methylprednisolone was administered at an initial dose of 64 mg/day, which was reduced to 8 mg/day and was eventually maintained at 4–8 mg/day.

We collected the baseline data of the donors, including age, sex, body mass index (BMI), history of diabetes, hypertension, cause of death, SCr level, and estimated glomerular filtration rate (eGFR) at admission and before procurement. We also collected the following baseline data of the recipients: age, sex, BMI, history of diabetes and hypertension, cause of ESRD, duration of dialysis, cold ischemia time (CIT), warm ischemia time (WIT), panel-reactive antibody (PRA) ≥ 20%, number of human leukocyte antigen (HLA) mismatches, induction therapy, and clinical outcomes. pre-transplant biopsy is not regularly performed for all AKI donor kidneys in our center. It is only being done as a last resort when the preoperative assessment of the deceased-donor kidney including surgeon appraisal, clinical parameters, and machine perfusion characteristics can not be determinative.

### Clinical outcomes and statistical analysis

The primary endpoints of this study were patient and allograft survival and renal function at different time points (1 week, 1 month, 6 months, and 1 year after kidney transplant [KT]). The secondary endpoints included the development of DGF, which was defined as a serum creatinine ≥ 400 µmol/L or required dialysis in the first week after KT [3, 22]. In addition, we compared allograft outcomes, including DGF, acute rejection, renal function, and patient and graft survival rates between the AKI and non-AKI groups and the pediatric and adult groups. The Chronic Kidney Disease Epidemiology Collaboration equation or Modified Schwartz formula (for pediatric) was used to calculate the eGFR [23, 24].

Continuous variables are presented as median (interquartile range [IQR]) or the mean ± SD and were compared using the Mann–Whitney U test/Kruskal–Wallis H test (for non-normally distributed variables) or Student’s t-test. Frequencies (percentages) were used for categorical data and were compared using chi-square tests or Fisher's exact test. Graft survival was estimated using the Kaplan–Meier method and compared among groups using the log-rank test. Logistic regression analysis was performed to predicting DGF. Cox proportional hazard regression analysis of risk factors for death-censored graft survival and patient survival. The multivariate analysis included variables that were statistically significant (p < 0.05) in Univariate Analysis and other clinically significant factors. For the inference testing, a two-sided p-value < 0.05 was considered statistically significant. Analyses were conducted using SPSS, version 26.0 (IBM Corp, Armonk, NY, USA).

## Results

### Baseline donor and recipient characteristics

The pediatric study cohort included 154 transplant recipients, including 41 (26.6%) in the AKI group and 113 (73.4%) in the non-AKI group. Pediatric donor kidneys are prioritized for use by children's receptors under our country's existing legislation, while in some situations adult receptors may also be used. However, because there are few pediatric ESRD patients on the waiting list, young and middle-aged adults are the majority recipients of pediatric deceased donor kidney in our center. Deceased donors with AKI were slightly older than those in the non-AKI group (9.70 ± 5.3 years vs. 7.11 ± 5.56 years; p = 0.006) and included fewer male patients (61.0% vs. 68.1%; P = 0.406); trauma was the most common cause of death in this study cohort. In this study cohort, 17, 13, and 11 patients had AKI classified as KDIGO stage 1, 2, and 3, respectively (Table 1).

**Table 1:** Demographic and clinical characteristics of deceased donors and recipients

	Pediatric donor cohort		p-value	Adult donor cohort		p-value
	AKI group (n = 41)	Non-AKI group (n = 113)		AKI group (n = 218)	Non-AKI group (n = 368)	
Donor						
Age, years, mean (SD)	9.70 ± 5.43	7.11 ± 5.56	0.006	46.29 ± 11.71	48.21 ± 11.51	0.067
Male, n (%)	25 (61.0)	77 (68.1)	0.406	187 (85.8)	308 (83.7)	0.501
Weight, kg, mean (SD)	29.67 ± 12.51	28,15 ± 21.02	0.075	66.75 ± 9.59	66.01 ± 11.54	0.290
BMI, mean (SD)	19.29 ± 4.39	18.35 ± 4.85	0.276	23.65 ± 3.20	23.37 ± 4.06	0.068
History of hypertension, n (%)	0	1(0.9)	1.000	58 (26.6)	88 (23.9)	0.466
History of diabetes, n (%)	0	0	1.000	12 (5.5)	21 (5.7)	0.918
Cause of death, n (%)			< 0.01			0.048
Trauma	27 (65.9)	46 (40.7)		78 (35.8)	124 (33.7)	
Cerebrovascular accident	14 (34.1)	37 (32.7)		136 (62.4)	221 (60.1)	
Other	0	30 (26.5)		4 (1.8)	23 (6.2)	
By KDIGO stage, n (%)			< 0.01			< 0.01
Stage 1	17 (41.5)	NA		86 (39.4)	NA	
Stage 2	13 (31.7)	NA		80 (36.7)	NA	
Stage 3	11 (26.8)	NA		52 (23.9)	NA	
SCr, µmol/L, median (IQR)						
At admission	39.29 (30.10–59.90)	41.00 (30.50–66.40)	0.678	71.40 (58.20–87.70)	70.60 (56.15–88.00)	0.293
Terminal	89.50 (63.80–148.30)	40.20 (26.07–54.00)	< 0.01	163.45 (114.85–213.75)	71.20 (53.55–88.00)	< 0.01
GFR, mL/min*1.73 m^2^, median (IQR)						
At admission	178.24 (134.26–187.05)	167.14 (136.48–193.76)	0.066	109.54 (85.54–118.09)	108.87 (87.91–121.61)	0.675
Terminal	89.53 (52.65–140.18)	172.86 (149.54–209.07)	< 0.01	40.00 (29.21–61.80)	105.32 (86.65–120.53)	< 0.01
Recipients						
Age, years, mean (SD)	35.93 ± 12.38	33.72 ± 13.19	0.387	39.46 ± 10.31	39.85 ± 10.21	0.549
Male, n (%)	28 (68.3)	71 (62.8)	0.573	161 (73.9)	267 (72.6)	0.732
BMI, mean (SD)	21.53 ± 3.04	21.97 ± 11.59	0.063	22.43 ± 3.98	22.54 ± 3.52	0.590
Diabetes, n (%)	0	3 (2.7)	0.565	16 (7.3)	26 (7.1)	0.901
Hypertension, n (%)	31 (75.6)	96 (85.0)	0.178	188 (86.2)	232 (87.8)	0.591
Days on dialysis, years, mean (SD)	1.44 ± 1.46	1.93 ± 2.81	0.822	2.11 ± 1.89	2.00 ± 2.14	0.074
Cause of ESRD, n (%)			0.322			0.179
Diabetes	0 (0)	2 (1.8)		10 (4.6)	13 (3.5)	
Glomerulonephritis	38 (92.7)	105 (92.9)		186 (85.3)	332 (90.2)	
Hypertension	0	3 (2.7)		17 (7.8)	14 (3.8)	
Other	3 (7.3)	3 (2.7)		5 (2.3)	9 (2.4)	
Transplant						
WIT, min, mean (SD)	3.94 ± 14.15	1.45 ± 3.07	0.291	1.96 ± 4.51	1.99 ± 5.04	0.686
CIT, hours, mean (SD)	10.29 ± 3.11	10.92 ± 3.77	0.401	10.45 ± 2.97	10.66 ± 3.30	0.056
PRA I ≥ 20, n (%)	2 (4.9)	5 (4.4)	0.905	14 (6.4)	22 (6.0)	0.829
PRA II ≥ 20, n (%)	2 (4.9)	7 (6.2)	0.758	4 (1.8)	12 (3.3)	0.306
HLA mismatches, mean (SD)	1.68 ± 1.11	1.72 ± 0.93	0.907	1.61 ± 1.03	1.67 ± 1.00	0.522
Induction immunosuppression (n, %)			0.180			0.015
Basiliximab	15 (36.6)	28 (24.8)		57 (26.1)	141 (38.3)	
ATG	23 (56.1)	66 (58.4)		128 (58.8)	180 (48.9)	
No-use	3 (7.3)	19 (16.8)		33 (15.1)	49 (13.3)	

*BMI* body mass index, *WIT* warn ischemia time, *CIT* cold ischemia time, *PRA* panel-reactive antibodies, *HLA* human leukocyte antigen, *ATG* anti-human T lymphocyte rabbit immunoglobulin, *DGF* delayed graft function, *PNF* primary nonfunction, *SCr* serum creatinine, *eGFR* estimated glomerular filtration rate, *NA* not applicable

There were 586 adult KT during the study period (218 [37.2%] in the AKI group and 368 [62.8%] in the non-AKI group). Cerebrovascular accidents were the most common cause of death in this cohort. Most of the 218 deceased donors with AKI were classified as stage 1 (86 [39.4%]), followed by stage 2 (80 [36.7%]), and stage 3 (52 [23.9%]). A significant statistical difference was observed in the induction immunosuppression regimen in the adult cohort, which may be attributed to the fact that DGF was more likely to occur, and anti-human T lymphocyte rabbit immunoglobulin (ATG) will tend to be selected for preoperative induction immunosuppression therapy. The other donor clinical indicators and recipients’ baseline characteristics were similar (Table 1).

### Clinical outcomes

**Fig. 1:**
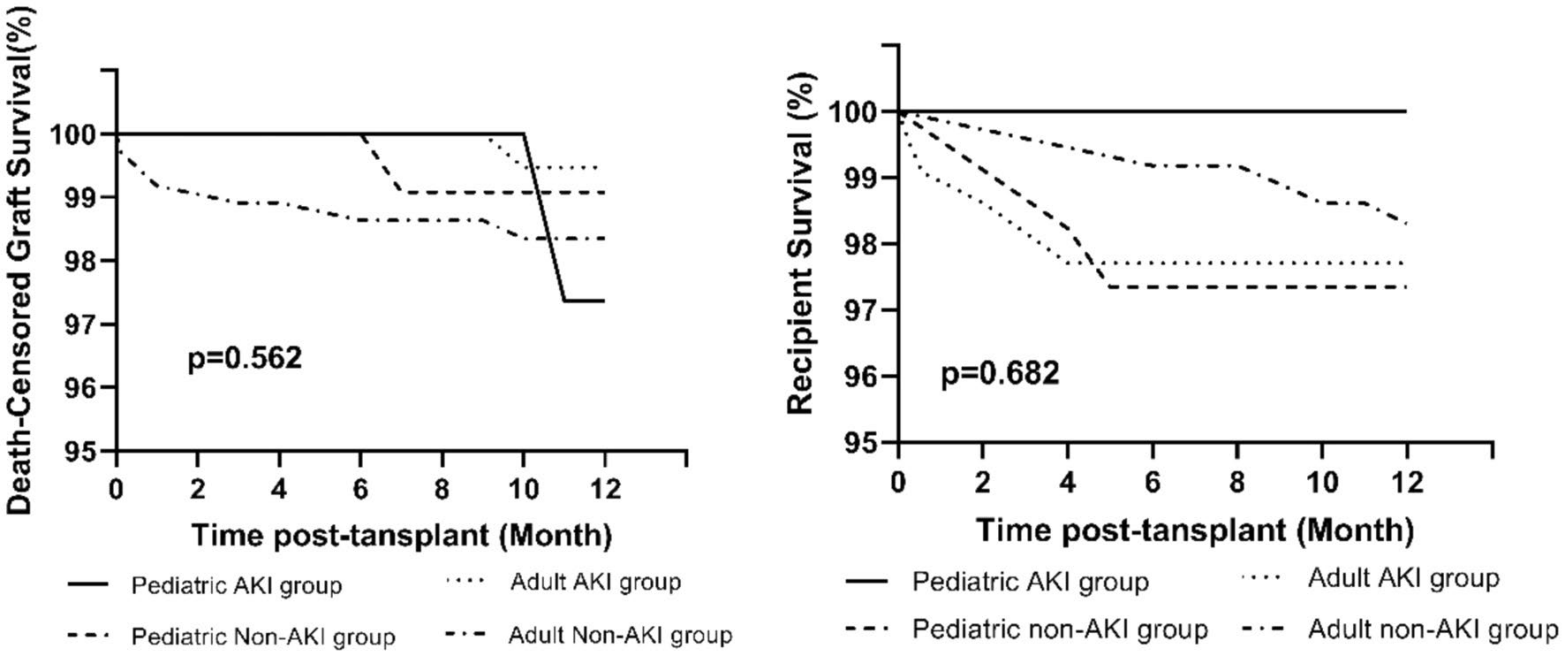
Kaplan–Meier curve illustrating patient and graft survival in the cohort study of kidney transplant recipients. Group comparisons were performed using the log-rank tests

**Fig. 2:**
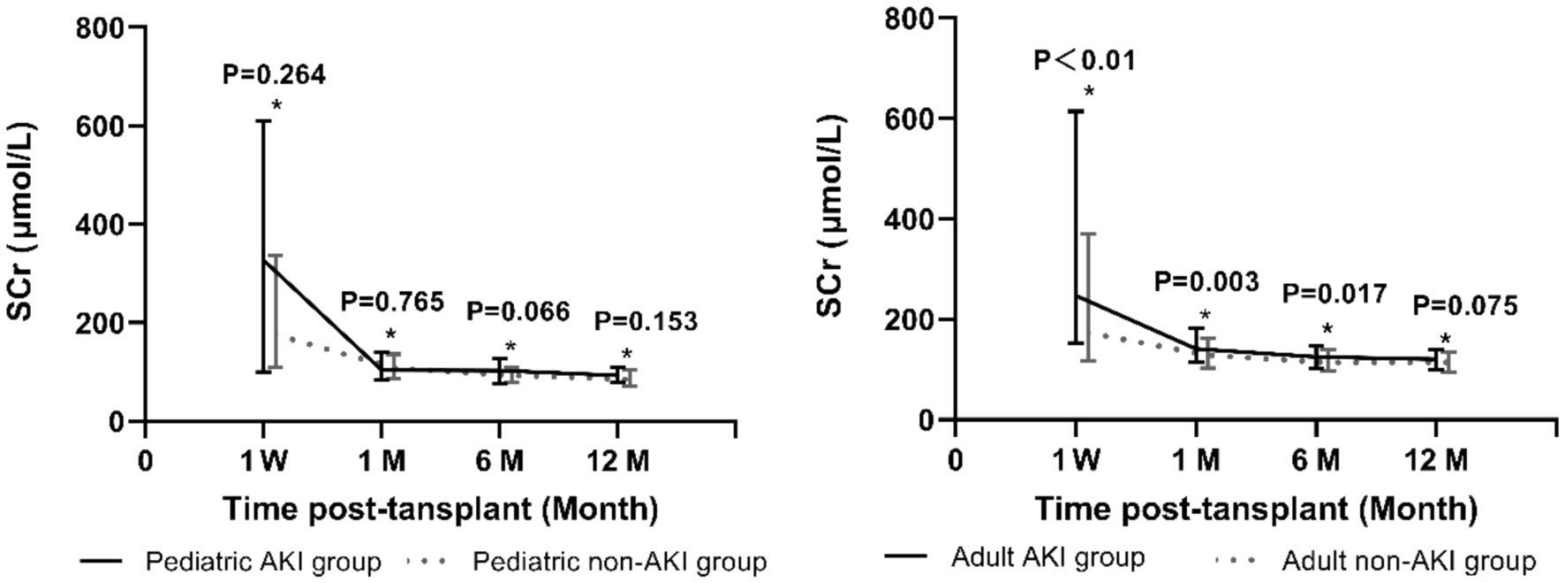
Postoperative trend of SCr

In the pediatric study cohort, the comparison of death-censored graft survival and patient survival at 1 year showed no significant differences (Table 2; Fig. 1). The 12-month death-censored graft survival rates were 97.4% in the AKI group and 99.1% in the non-AKI group (p = 0.472). The 12-month patient survival was 100% in the AKI group and 97.3% in the non-AKI group (p = 0.295). As shown in Fig. 2, the trend of post-renal transplant SCr levels rapidly recovered to normal levels in both groups. No significant difference was observed between the two groups, even in the first week post-transplant, which demonstrates the powerful recovery potential of pediatric AKI kidneys. The incidence of DGF was higher in the recipients of allografts with AKI than in those without AKI (34.1% vs. 16.8%, p = 0.021). There was no difference in the rate of acute rejection episodes in the first year post-transplant in the study cohort (p = 0.654). Results in Clinical Outcomes which were stratified by AKI severity manifest the incidence of DGF was associated with donor AKI status (p = 0.039), but unrelated with AKI stage in the pediatric cohort (p = 0.412). There was no difference in the rate of acute rejection episodes in the first year post-transplant (p = 0.461), death-censored graft survival (p = 0.402), and patient survival at 1 year (0.777). The trend of SCr levels post-renal transplant recovered to normal levels in pediatric cohort rapidly (Table 3).

**Table 2:** Clinical outcomes

	Pediatric cohort		p-value	Adult cohort		p-value
	AKI group (n = 41)	Non-AKI group (n = 113)		AKI group (n = 218)	Non-AKI group (n = 368)	
Follow-up time (years)	11.68 ± 1.23	11.42 ± 1.77	0.534	11.17 ± 2.20	11.58 ± 1.30	0.254
DGF, n (%)	14 (34.1)	29 (16.8)	0.021	84 (38.5)	64 (17.4)	< 0.01
Acute rejection first year	7 (17.1)	16 (14.2)	0.654	29 (13.3)	47 (12.8)	0.853
SCr (µmol/L)						
1 week	326.70 (100.35–609.85)	175.40 (109.20–336.15)	0.264	246.70 (153.05–614.03)	172.95 (116.75–369.95)	< 0.01
1 month	103.80 (83.70–140.05)	108.10 (86.40–136.35)	0.765	141.20 (115.00–182.05)	128.40 (102.80–161.60)	0.003
6 months	103.20 (76.35–127.40)	93.20 (78.30–108.00)	0.066	125.30 (102.30–146.85)	113.90 (97.00–138.70)	0.017
12 months	93.00 (79.00–109.75)	85.10 (71.05–104.10)	0.153	120.00 (100.55–139.00)	114.00 (95.00–134.48)	0.075
eGFR (mL/min*1.73 m^2^)						
1 week	16.49 (9.79–68.37)	38.37 (19.35–64.66)	0.308	24.38 (8.68–45.24)	38.48 (15.55–62.39)	< 0.01
1 month	70.92 (48.34–94.53)	68.87 (54.19–87.80)	0.748	48.35 (36.34–63.94)	55.72 (41.84–72.47)	0.005
6 months	73.15 (56.91–97.03)	83.15 (70.13–99.17)	0.148	57.32 (46.65–74.60)	63.87 (50.42–78.65)	0.025
12 months	86.62 (71.00–102.64)	87.54 (77.9–106.69)	0.226	63.21 (48.34–78.14)	65.05 (53.76–78.55)	0.142
1-year clinical outcome						
Graft loss incident, n (%)	1 (2.4)	1 (0.9)	0.452	1 (0.5)	6 (1.6)	0.207
Death-censored graft survival rate, (%)	97.4	99.1	0.472	99.5	98.3	0.217
Patient death incident, (%)	0	3 (2.7)	0.565	5 (2.3)	6 (1.6)	0.568
Patient survival, (%)	100	97.3	0.295	97.7	98.3	0.544

*DGF* delayed graft function, *SCr* serum creatinine, *eGFR* estimated glomerular filtration rate

**Table 3:** Clinical outcomes stratified by AKI severity in pediatric cohort

	Stage 1 (n = 17)	Stage 2 (n = 13)	Stage 3 (n = 11)	non-AKI (n = 113)	p-value
DGF, n (%)	6 (35.3)	3 (23.1)	5 (45.5)	19 (16.8)	0.039
AR, n (%)	2 (11.8)	1 (7.7)	4 (36.4)	16 (14.2)	0.461
SCr, µmol/L, median (IQR)					
1 week	411.34 (200.83–621.87)	287.53 (88.55–486.50)	310.13 (159.70 (460.55)	299.16 (239.00–359.32)	0.299
1 month	117.45 (93.47–141.43)	106.74 (90.95–122.53)	112.45 (87.24–137.66)	126.15 (108.53–143.78)	0.988
6 months	157.66 (81.65–233.67)	80.25 (59.56–100.94)	105.65 (77.86–133.44)	95.62 (87.35–103.88)	0.026
12 months	118.98 (78.21–59.74)	83.64 (71.69–95.59)	110.96 (64.77–157.16)	91.63 (83.37–99.91)	0.129
1-year clinical outcome					
Graft loss incident, n (%)	1 (5.9)	0 (0)	0 (0)	1 (0.9)	0.228
Death-censored graft survival rate, (%)	94.1	100	100	99.1	0.402
Patient death incident, n (%)	0 (0)	0 (0)	0 (0)	3 (2.7)	0.392
Patient survival, (%)	100	100	100	97.3	0.777

*DGF* delayed graft function, *SCr* serum creatinine, *eGFR* estimated glomerular filtration rate

In the adult cohort, no significant differences were noted in death-censored graft survival (at 1 year, 99.5% [AKI group] vs. 98.3% [non-AKI group], p = 0.217) or patient survival (at 1 year, 97.7% [AKI group] vs. 98.3% [non-AKI group], p = 0.544) between the two groups (Table 2, Fig. 1). SCr at 1 week, 1 month, and 6 months was significantly higher in the AKI group. The downward trend in SCr was slower in the AKI group than in the non-AKI group; however, after one year, there was no statistically significant difference in SCr levels between the two groups (Fig. 2). The incidence of DGF was higher in recipients of allografts with AKI than in those of allografts without AKI (38.5% vs. 17.4%, p < 0.01) and tended to increase with the AKI stage (31.4%, 32.5%, and 59.6%, for stage 1, 2, and 3 AKI groups, respectively, p < 0.01). No significant differences were observed between the two groups in terms of acute rejection episodes (13.3% [AKI group] vs. 12.8% [non-AKI group], p = 0.853).

Deceased donor AKI status itself did not affect death-censored graft survival, with a p-value of 0.442 (95%CI: 0.112–2.599). The overall death-censored graft survival rate was 97.7% (AKI group) vs. 97.5% (non-AKI group) at 1 year (p = 0.435). The overall patient survival rate was 98.1% (AKI group) vs. 98.1% (non-AKI group) at 1 year (p = 0.993). No significant differences were observed among the four groups in terms of patient survival (p = 0.682) and death-censored graft survival (p = 0.562). Due to the small cardinal number of pediatric AKI group, the death-censored graft survival rate was appeared to be lower at 1 year when there was one instance of graft loss (because of a severe infection). In the early post-transplantation period, patient and graft survival rates remained acceptable between groups, with a mean follow-up period of 11.44 ± 1.68 months (Fig. 1).

### Regression analysis for DGF, death-censored graft survival and patient survival

The presence of donor AKI was an independent risk factor for DGF at multivariate logistic regression model of the total cohort (p = 0.040, 95%CI:1.025–2.834). The history of diabetes in the donors, SCr level at Terminal, warm and cold ischemia time also had a negative effect on DGF (Table 4). In the Cox proportional hazard regression analysis, the presence of donor AKI and the severity of the AKI were not significantly associated with death-censored graft survival and patient survival. DGF after kidney transplantation adversely affected patient survival (p = 0.002, HR = 6.189, 95%CI: 1.905–20.106) and death-censored graft survival (p = 0.010, HR = 7.096, 95%CI:1.598–31.505). Acute rejection events had a negative influence on death-censored graft survival (p = 0.001, 95%CI: 2.339–34.933) (Table 5).

**Table 4:** logistic regression analysis of main variables predicting DGF

	Univariate analysis		Multivariate analysis	
	OR (95%CI)	p-value	OR (95%CI)	p-value
Donor				
Male	1.769 (1.100–2.845)	0.019	1.226 (0.723–2.078)	0.450
Age	1.000 (0.991–1.009)	0.977		
Weight	1.008 (0.999–1.017)	0.069		
BMI	1.047 (1.008–1.088)	0.018	1.002 (0.958–1.048)	0.928
History of hypertension	1.363 (0.911–2.040)	0.132		
History of diabetes	2.383 (1.169–4.855)	0.017	2.576 (1.165–5.692)	0.019
SCr At admission	1.016 (1.009–1.023)	< 0.01	1.009 (0.999–1.019)	0.069
SCr At terminal	1.009 (1.007–1.012)	< 0.01	1.004 (1.000–1.009)	0.050
AKI	2.919 (2.067–4.121)	< 0.01	1.704 (1.025–2.834)	0.040
Stage 1	1.558 (0.990–2.450)	0.055		
Stage 2	1.476 (0.918–2.373)	0.108		
Stage 3	4.892 (2.875–8.325)	< 0.01	1.636 (0.692–3.868)	0.043
Recipients				
Age	0.997 (0.982–1.012)	0.705		
Male	1.299 (0.886–1.904)	0.181		
BMI	1.017 (0.988–1.045)	0.251		
History of diabetes	1.274 (0.654–2.484)	0.477		
History of Hypertension	0.938 (0.580–1.517)	0.794		
Days on dialysis	1.086 (1.010–1.167)	0.025	1.078 (0.998–1.165)	0.055
Transplant				
WIT	1.039 (1.006–1.073)	0.020	1.041 (1.006–1.077)	0.023
CIT	1.074 (1.019–1.131)	0.007	1.080 (1.019–1.144)	0.010
PRAI	1.532 (0.791–2.968)	0.206		
PRAII	1.776 (0.771–4.091)	0.177		
HLA mismatches	0.922 (0.780–1.090)	0.343		

*OR* odds ratio

**Table 5:** Cox proportional hazard regression analysis of risk factors for death-censored graft survival and patient survival

	Death-censored graft survival				Patient survival			
	Univariate analysis		Multivariate analysis		Univariate analysis		Multivariate analysis	
	HR (95% CI)	P	HR (95% CI)	P	HR (95% CI)	P	HR (95% CI)	P
Donor								
Male	28.035 (0.023–34,659.316)	0.359			1.426 (0.319–6.371)	0.642		
Age	0.996 (0.964–1.029)	0.813	1.016 (0.965–1.069)	0.545	0.992 (0.966–1.018)	0.542	0.982 (0.942–1.023)	0.382
Weight	0.988 (0.960–1.017)	0.407	0.973 (0.927–1.023)	0.283	1.002 (0.975–1.028)	0.909	1.009 (0.973–1.047)	0.619
BMI	0.915 (0.793–1.055)	0.222			1.031 (0.918–1.159)	0.603		
History of hypertension	1.117 (0.232–5.376)	0.891			1.076 (0.300–3.858)	0.910		
History of diabetes	0.046 (0–60,197.894)	0.669			3.638 (0.814–16.255)	0.091		
SCr At admission	1.005 (0.982–1.030)	0.665			1.023 (1.008–1.038)	0.003		
SCr At terminal	0.996 (0.984–1.008)	0.492			1.004 (1.000–1.007)	0.036		
AKI	0.540 (0.112–2.599)	0.442	0.344 (0.064–1.846)	0.213	1.048 (0.351–3.127)	0.933	0.603 (0.183–1.988)	0.406
Stage 1	0.773 (0.097–6.177)	0.808			1.034 (0.231–4.621)	0.965		
Stage 2	0.889 (0.111–7.108)	0.912			1.178 (0.264–5.263)	0.830		
Stage 3	0.044 (0.000–1443.619)	0.555			0.842 (0.110–6.434)	0.868		
Recipients								
Age	0.833 (0.208–3.329)	0.796	1.045 (0.980–1.115)	0.178	1.031 (0.981–1.083)	0.228	1.041 (0.987–1.099)	0.137
Male	1.021 (0.962–1.085)	0.492			34.623 (0.267–4492.826)	0.153		
BMI	1.008 (0.914–1.113)	0.867	1.000 (0.871–1.149)	0.996	1.010 (0.943–1.083)	0.768	0.991 (0.871–1.126)	0.885
History of diabetes	2.040 (0.255–16.309)	0.502			1.225 (0.160–9.364)	0.845		
History of hypertension	1.297 (0.162–10.366)	0.807			0.968 (0.217–4.325)	0.966		
Days on dialysis	0.952 (0.677–1.339)	0.778	0.938 (0.672–1.309)	0.705	0.953 (0.724–1.253)	0.730	0.933 (0.711–1.223)	0.615
Transplant								
WIT	1.007 (0.913–1.11)	0.892	0.982 (0.886–1.088)	0.724	1.037 (1.008–1.067)	0.011	1.017 (0.984–1.050)	0.318
CIT	0.901 (0.713–1.139)	0.382	0.861 (0.677–1.095)	0.223	0.998 (0.843–1.181)	0.981	0.968 (0.821–1.141)	0.698
PRAI	4.549 (0.945–21.898)	0.059			0.045 (0–887.290)	0.540		
PRAII	0.047 (0–355,677.13)	0.705			0.047 (0–17,233.146)	0.640		
HLA mismatches	1.256 (0.642–2.456)	0.505	1.367 (0.664–2.813)	0.396	0.914 (0.544–1.534)	0.733	1.007 (0.583–1.742)	0.979
DGF	3.976 (1.068–14.806)	0.040	7.096 (1.598–31.505)	0.010	5.680 (1.903–16.948)	0.002	6.189 (1.905–20.106)	0.002
AR	8.548 (2.295–31.845)	0.001	9.039 (2.339–34.933)	0.001	2.653 (0.832–8.460)	0.099	2.195 (0.621–7.759)	0.223
Induction immunosuppression (n, %)								
Basiliximab	0.250 (0.031–1.998)	0.191			0.555 (0.155–1.991)	0.367		
ATG	1.129 (0.303–4.207)	0.856			1.598 (0.535–4.769)	0.401		
No-use	3.039 (0.760–12.153)	0.116			1.016 (0.227–4.538)	0.984		

*HR* hazard ratio

## Discussion

The presence of AKI in organ donors appears to impact the willingness to accept and transplant donor kidneys in China [25]. The incidence rate of AKI ranges from 25 to 52% within pediatric intensive care units [26, 27], and to augment the donor pool with systematic analysis of kidney transplants from pediatric donors with AKI, we found that although the incidence of DGF was higher in recipients of allografts with AKI, deceased donor AKI status itself did not affect death-censored graft and recipient survival. Patients with kidney transplants from a pediatric AKI donor had a superior recovery of allograft function. This result may help decrease the number of discarded pediatric AKI kidneys and utilize this donor pool to minimize waiting-list-related mortality.

AKI is usually secondary to prerenal factors (e.g., hypovolemia, cardiac insufficiency), neurohormonal mechanisms, rhabdomyolysis, or the use of nephrotoxic agents in critically ill patients [7, 11]. The cause of AKI has long been debated, and no specific therapies have emerged that can expedite recovery or attenuate AKI [7, 28]. AKI is often considered a reversible functional renal impairment and is mainly characterized by acute tubular necrosis (ATN) [3, 29].

The literature on AKI recovery patterns, according to the Acute Disease Quality Initiative (ADQI) definition, has found a high rate of early recovery and transient AKI. The cumulative incidence of renal recovery increased progressively from 25% on day 5 to 41% on day 10. However, up to 40% of patients with AKI do not recover by day 7, and 38% of AKI cases persistent beyond 7 days can be defined as acute kidney disease [30]. Some hypothesized that AKI and CKD may have interconnections [31] and can be weakened in the renal recipients’ internal environments, especially considering that kidneys procured from pediatric deceased donors may have greater repair potential because the donors are younger and have fewer underlying diseases or comorbidities, donor AKI can activate body repair processes and initiate ischemic preconditioning, and complete perioperative management can be beneficial for graft function repair in the recipient [4, 16].

Prior research on the utilization of pediatric deceased donors with AKI was limited. Jiang et al. [25] found that transplants procured from pediatric AKI donors have a comparable renal function and an excellent patient and graft survival rate, but they found a similar incidence of DGF, which is different from our study. In contrast, our study consists with another study includes very small (≤ 15 kg) pediatric donors with AKI and found that AKI can impact early post-transplant kidney graft function (e.g., the rate of DGF), but it did not increase the risk of early graft loss or decreased renal function in the long term [18]. A national study of pediatric KT recipients from a donor with AKI found that donor AKI status or increased peak and terminal creatinine levels do not affect the rate of DGF in pediatric KT recipients [17]. They also demonstrated that younger donors can be a protective factor for renal recovery, which is consistent with our finding that pediatric AKI donors have a superior recovery of allograft function.

In the adult cohort, the incidence of DGF was higher in recipients of allografts with AKI than in those without AKI (38.5% vs. 17.4%, p < 0.01) and tended to increase with the AKI stage, which is consistent with previous studies [6, 32]. Lui et al. [3] and Kwon et al. [33] reported that deceased donor AKI status did not affect death-censored graft survival and patient survival in an adult study cohort with a similar survival rates, and it can provide favorable graft functions for the later enginery, which is consistent with our study, but they did not analyze the trend of SCr postoperatively. In this study, we found that the downward trend in SCr was slower in the AKI group than in the non-AKI group, but after one year, there was no statistically significant difference in SCr levels between the two groups.

Denic et al. [34] analyzed both non-sclerotic glomeruli (NSG) and globally sclerotic glomeruli (GSG) using computed tomography scans and pathological biopsy before transplantation in living kidney donors and found that the number of NSG decreases with age, while GSG and the missing glomeruli increase with age, which is approximately proportional to the decline in GFR. In autopsy series [35, 36], the number of nephrons that decline with age is consistently evident. Considering that kidneys procured from pediatric deceased donors may have greater repair potential because the donors are younger with more NGS, fewer underlying diseases, or comorbidities [17, 18], and previous studies have demonstrated that younger donors can be a protective factor for renal recovery, even in pediatric AKI donors [17], pediatric AKI renal transplantation had a comparable clinical outcome and superior recovery of allograft function, tending to be superior to adult deceased donors with or without AKI. Therefore, the transplant community should obtain a new perspective on this type of organ pool and its potential use.

## Strengths and limitations

Our study has several strengths. This was a large-scale clinical cohort study. Previous research [18, 25] has been limited by less observation time or small sample sizes, particularly for the number of pediatric deceased donors with stage 2 and 3 AKI. Second, we confirmed our research by comparing early clinical outcomes between the pediatric and adult cohorts in detail. Several limitations of our study should be considered, including its retrospective, single-institution cohort nature, and within the period of this research there are very few death-censored graft loss or death events, so the censored data and selection bias were inevitable due to the evolution of the reliability and validity of the analysis process. Second, organ procurement decisions are multifactorial, but we have not analyzed the reason for the AKI-associated discard rate or many other factors involved in KT decisions. Third, the mean follow-up time of this study was 11.44 ± 1.68 months, and further prospective studies with long-term kidney allograft outcomes and larger study cohorts are required to strengthen this conclusion.

## Conclusions

Kidney transplantation from deceased donors with AKI remains controversial. Prior research on the utilization of donors with pediatric AKI was limited. Compared with non-AKI pediatric donor kidneys, we found that AKI pediatric donor kidneys not only have similar excellent clinical outcomes but also show a comparable recovery speed, which indicates the superior recovery of allograft function of pediatric kidneys. However, in the adult cohort of our study, the downward trend in SCr level was substantially slower in the AKI group than that in the non-AKI group. However, at 1 year post-transplant, there was no statistically significant difference in the graft survival rate, patient survival, and AR between the adult AKI and non-AKI groups. The transplant community should utilize this donor pool, but further long-term prospective studies with larger cohorts are required to strengthen this conclusion. Our data showed that deceased donor AKI status had a negative effect on the DGF rate; it did not affect death-censored graft survival and recipient survival. Therefore, the transplant community should utilize this donor pool to minimize waiting-list-related mortalities. Future prospective studies with long-term kidney allograft outcomes and larger study cohorts are warranted if pediatric deceased donors with AKI are widely adopted.

## Data Availability

The data sets used and/or analyzed during the current study are available from the corresponding author on reasonable request. Dr. Gongbin Lan, e-mail address: langongbin@csu.edu.cn.
